# Importance of gradual induction in oral anticoagulation therapy in a patient with heparin-induced thrombocytopenia with a left ventricular assist device: a case report

**DOI:** 10.1186/s44215-022-00006-1

**Published:** 2022-09-27

**Authors:** Kenichi Morimoto, Yasushi Yoshikawa, Yuichiro Kishimoto, Takeshi Onohara, Motonobu Nishimura

**Affiliations:** Division of Cardiovascular Surgery, Department of Surgery, Faculty of Medicine, Tottori University, 36-1 Nishi-cho, Yonago, Tottori, 683-8504 Japan

**Keywords:** HIT, iLVAD, Thrombosis

## Abstract

**Background:**

We encountered a case of a patient with heparin-induced thrombocytopenia (HIT) who developed multiple thromboses following implantable left ventricular assist device (iLVAD) implantation. We herein report this case along with a literature-based discussion on the timing of warfarin initiation for such patients.

**Case presentation:**

A 15-year-old boy was rushed to our hospital due to cardiogenic shock associated with multiple organ dysfunction. A detailed examination, including cardiac catheterization and right heart biopsy, provided a diagnosis of dilated cardiomyopathy, for which an iLVAD placement as a bridge to cardiac transplantation was scheduled after the transplant workup. Three days before the surgery, the patient’s platelet count decreased drastically from 110,000 to 40,000/µL, suggesting HIT, and his blood sample was sent to a specialized laboratory outside our hospital. The HIT antibody test came back positive during the iLVAD implantation using heparin. Thus, we chose argatroban over heparin for postoperative anticoagulation therapy, which was initiated 6 h after the surgery. The day after surgery, the patient was successfully weaned off mechanical ventilation and extubated. Oral anticoagulation therapy with warfarin was also initiated; however, on postoperative day 2, contrast-enhanced computed tomography revealed multiple thrombi in the left iliac artery, right iliac vein, and right jugular vein. We suspected hypercoagulation associated with the initiation of warfarin, prompting us to suspend warfarin and continue anticoagulation therapy with argatroban alone. On postoperative day 8, the patient’s platelet count increased to 130,000/µL despite not reaching the normal range and showed two consecutive increases; therefore, we resumed oral warfarin, concerning the efficacy of argatroban as an anticoagulant for iLVAD. Thromboembolism did not recur; the patient was discharged 50 days after the operation and is currently awaiting heart transplantation.

**Conclusions:**

We encountered a case of HIT type II in which multiple thromboses occurred following iLVAD implantation. If HIT antibodies are detected, it is crucial to initiate argatroban monotherapy and wait to start oral anticoagulation therapy with warfarin until platelet count has improved.

## Background

Heparin-induced thrombocytopenia (HIT) is a condition in which thrombocytopenia and thromboembolism are triggered due to platelet activation via immunological mechanisms [1, 2]. The frequency of HIT is reported to be high in patients with an implantable left ventricular assist device (iLVAD) [3, 4]. We encountered a case of a patient who suffered from cardiogenic shock associated with dilated cardiomyopathy. He was identified as being HIT antibody-positive during the iLVAD implantation surgery and developed multiple thromboses shortly after initiating oral anticoagulation therapy with warfarin, possibly due to warfarin-induced hypercoagulation.

## Case presentation

**Fig. 1:**
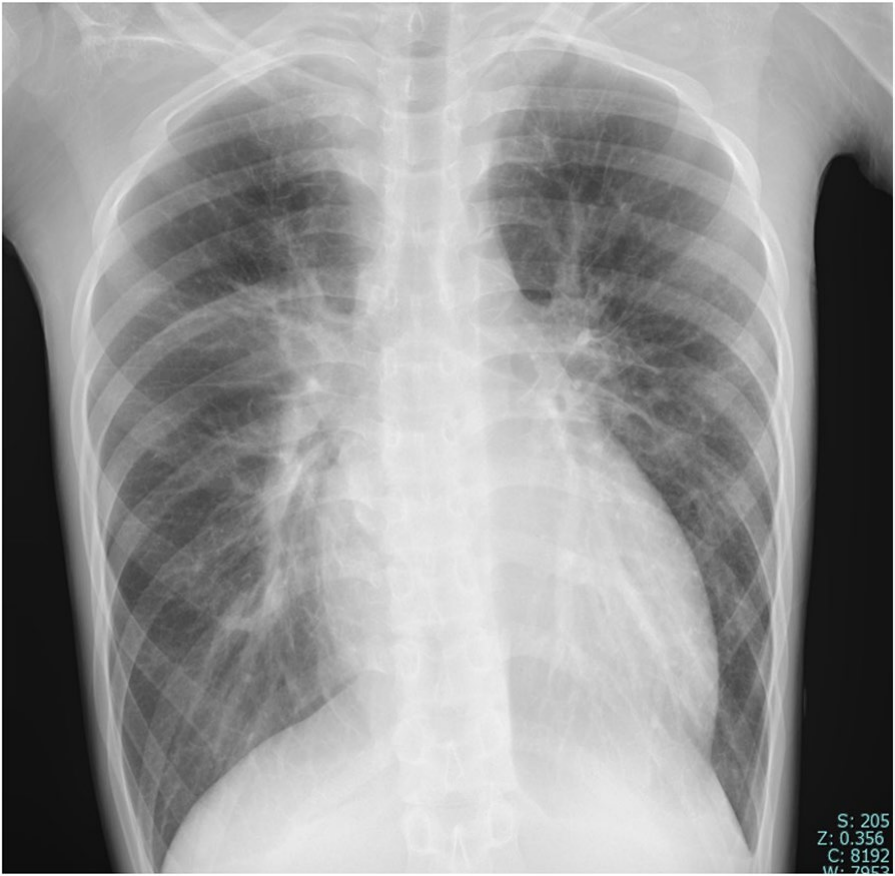
Chest X-ray at admission. The X-ray shows the enlargement of a cardiac shadow with a cardiothoracic ratio of 0.62, accompanied by severe pulmonary congestion

**Fig. 2:**
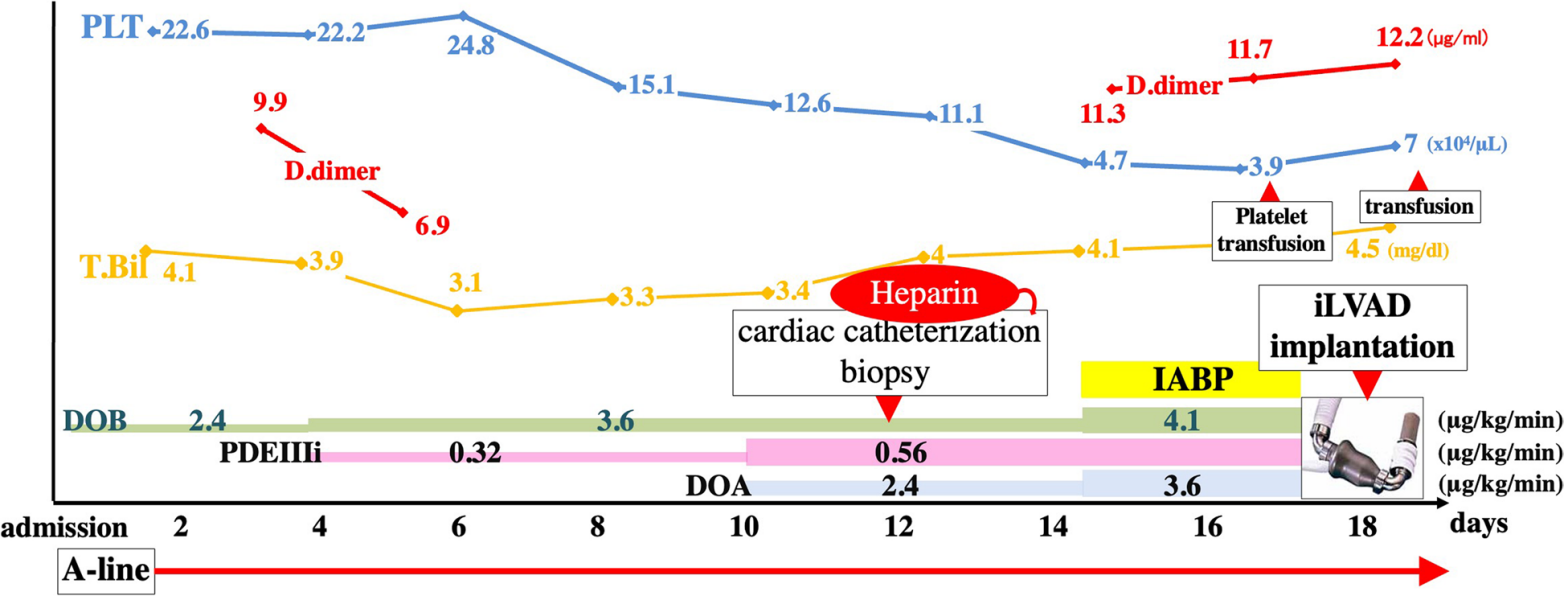
The clinical course prior to iLVAD surgery. Heparin had been administered immediately after admission through an arterial pressure line, as well as at the time of cardiac catheterization. A marked decrease in platelet count was observed between days 13 and 14. A-line: placement of the arterial pressure line, PLT: platelet count (/μL), T.Bil: Serum total bilirubin level (mg/dL), DOB: continuous dobutamine infusion (μg/kg/min), DOA: continuous dopamine infusion (μg/kg/min), PDE III i: continuous infusion of phosphodiesterase III inhibitor (μg/kg/min), IABP: placement of intra-aortic balloon pumping

**Fig. 3:**
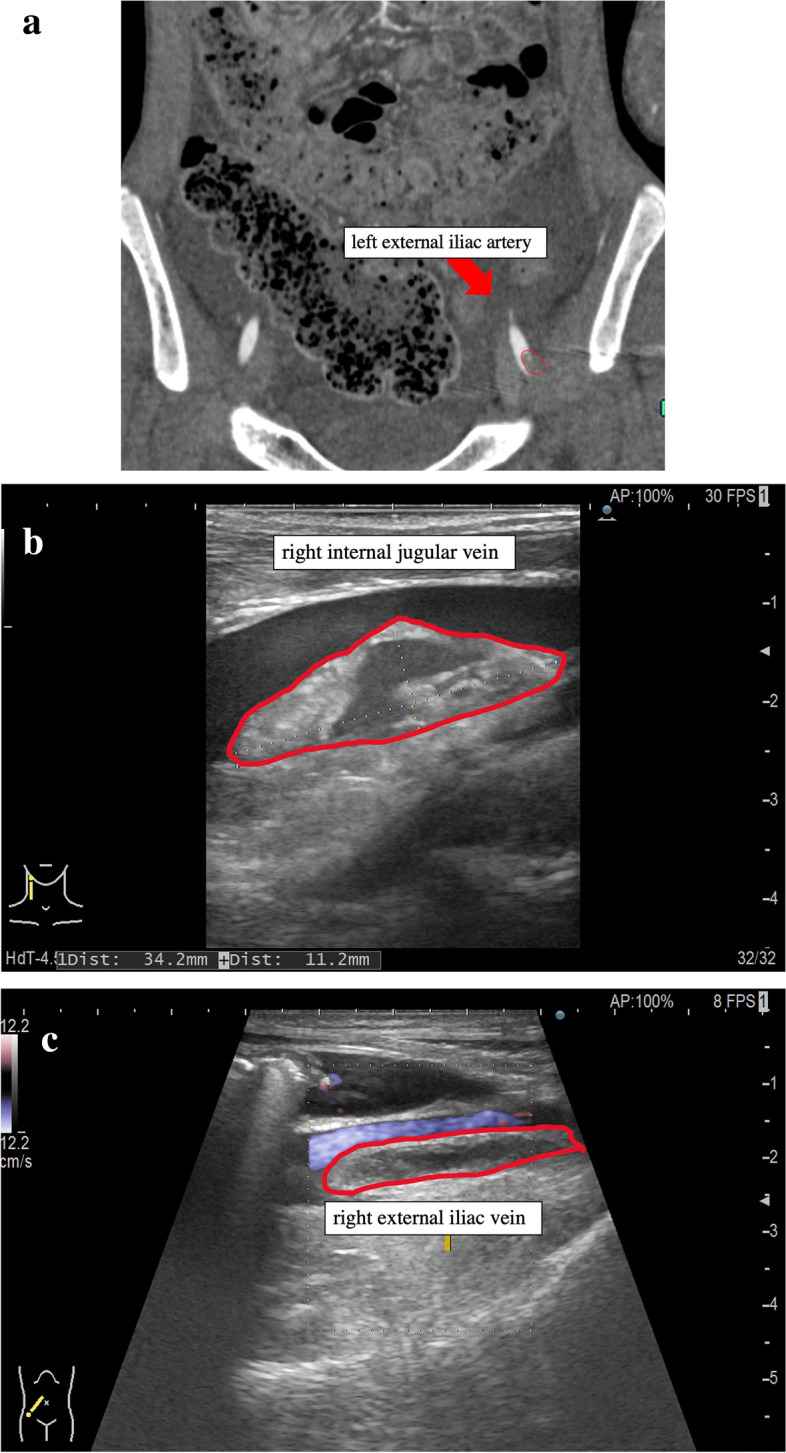
Detection of multiple thrombi by contrast-enhanced computed tomography (CE-CT) and venous ultrasonography. **a** Sagittal view of CE-CT. A thrombus is seen in the wall of the left external iliac artery (inside the red circle). **b** Venous ultrasonography of the right internal jugular vein. A thrombus is seen in the wall of the right internal jugular vein (inside the red circle). **c** Venous ultrasonography of the right external iliac vein. A thrombus is seen in the wall of the right external iliac vein (inside the red circle)

**Fig. 4:**
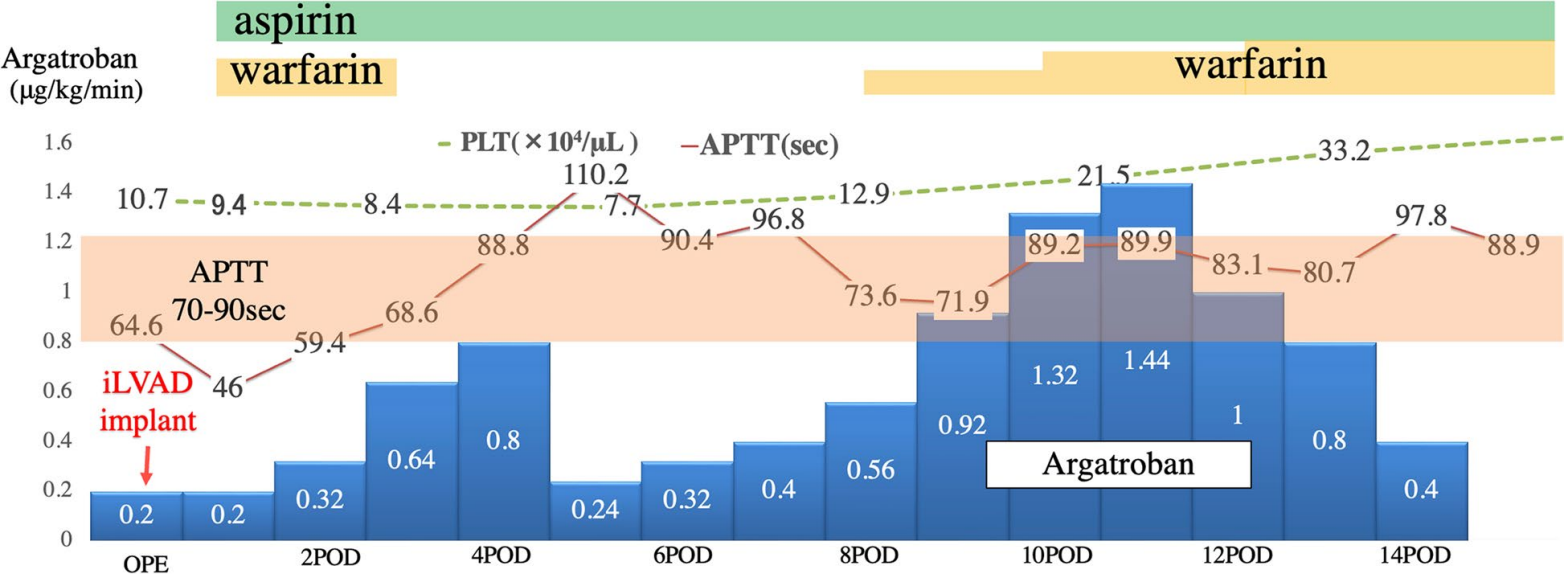
The course of postoperative anticoagulation. The dose of argatroban was controlled to the target APTT (70–90 s). The target range is shown in pink shadow. The oral anticoagulation therapy with warfarin was started on POD 1 and stopped on POD 2. After the platelet counts returned to the normal range, warfarin was restarted on POD8. PLT, platelet count; APTT, activated partial thromboplastin time; Sec, seconds

A 15-year-old boy was admitted to our hospital for dyspnea at rest. He was 170 cm tall and weighed 42.1 kg. His blood pressure and heart rate were 78/47 mmHg and 124 beats/min, respectively. Chest radiography revealed marked cardiomegaly (cardiothoracic ratio 0.62) and severe pulmonary congestion (Fig. 1). Blood test results revealed marked elevation of hepatic enzymes and serum bilirubin levels, total bilirubin, 4.1 mg/dL (normal < 1.5 mg/dL); aspartate aminotransferase, 1246 U/L (normal < 30 U/L); and alanine aminotransferase, 977 U/L (normal 23 U/L). The platelet count during admission was 220,000/µL. Serum brain natriuretic peptide level was also markedly elevated to 2974 pg/mL (normal < 18.4 pg/mL). Oliguria was observed with a serum creatinine level of 1.09 mg/dL and blood urea nitrogen of 53 mg/dL; however, urine volume was maintained at about 1500 mL/day with continuous intravenous administration of furosemide after admission. Echocardiography showed severe diffuse hypokinesis (left ventricular end-diastolic diameter 68 mm, left ventricular end-systolic diameter 66 mm, and left ventricular ejection fraction 7.1%) with severe mitral regurgitation and no aortic regurgitation. Cardiac catheterization showed mean pulmonary artery pressure of 36 mmHg, pulmonary wedge pressure of 28 mmHg, right atrial pressure of 14 mmHg, and cardiac output (cardiac index) of 1.94 L/min (1.25 L/min/m2) with Fick’s formula. Right ventricular endomyocardial biopsy was also performed simultaneously, and a definitive diagnosis of idiopathic dilated cardiomyopathy was made from the pathological findings. Cardiac transplantation was indicated, and the workup for transplantation was started at the same time. Since admission, continuous intravenous catecholamines had been initiated; however, we observed exacerbation of congestion and an increase in serum bilirubin level in the next several days. Circulation did not improve despite increasing the doses of catecholamines; therefore, on day 14 of hospitalization, we placed an intra-aortic balloon pump (IABP). Approval for cardiac transplantation was also obtained from the Cardiac Transplantation Committee of the Japanese Circulation Society; therefore, shortly after heart transplantation registration, we scheduled to implant an iLVAD (HeartMate II®). During IABP, deep venous thrombosis in the femoral vein was incidentally found, and heparin was initiated. Although the patient’s platelet counts drastically decreased to 39,000/µL on day 14 after hospitalization and a test for HIT antibodies was submitted, we considered that IABP could be the primary cause of thrombocytopenia and did not strongly suspect the possibility of HIT (Fig. 2). The patient’s hemodynamics gradually deteriorated, and we rushed to proceed with iLVAD implantation 3 days after the IABP initiation. Since the HIT antibody test results had not returned, we performed iLVAD surgery with heparin as an intraoperative anticoagulant. The results of the HIT antibody test came back positive (1.6 U/mL, normal < 1.0 U/mL) during the operation. Thus, 6 h after surgery, we initiated postoperative anticoagulation therapy with argatroban at a dose of 0.2 µg/kg/min and adjusted the dose to achieve an activated partial thromboplastin time of 70 − 90 s based on the guideline that showed the therapeutic range (i.e., 1.5–3.0 times the pre-argatroban baseline value but not exceeding 100 s). The day after surgery, the patient was successfully weaned off mechanical ventilation and extubated. Oral anticoagulation therapy with warfarin was also initiated at a low dose of 2 mg/day in addition to aspirin on postoperative day 1, and the patient’s platelet count was 91,000/µL. On postoperative day 2, multiple arterial and venous thrombi were found by contrast-enhanced computed tomography (CE-CT) and venous ultrasonography (Fig. 3), prompting us to discontinue warfarin. On day 8 after the surgery, the patient’s platelet count had improved to 130,000/µL; therefore, we re-initiated warfarin. We subsequently tapered the dosage of argatroban gradually. On postoperative day 14, the patient’s prothrombin time reached the therapeutic range, and we completely discontinued argatroban (Fig. 4). The repeated CE-CT and venous ultrasonography revealed the disappearance of the multiple thrombi, and the patient showed no thromboembolic events during the treatment course. On postoperative day 30, HIT antibody test results were negative. The patient was discharged without sequelae on day 50 and returned to school on day 100. Currently, he is awaiting heart transplantation.

## Discussion

HIT, a major adverse event associated with the use of heparin, is generally classified into two types. Type I is a non-immune form of thrombocytopenia in which platelet aggregation is triggered by the physicochemical properties of heparin. Although thrombocytopenia occurs within 2 days after heparin administration, the risk of thrombosis is low, and the thrombocytopenia is reversible. Contrarily, in type II, thrombocytopenia and thrombosis are triggered by an immune mechanism. In this pathology, heparin binds to platelet factor 4 (PF4), forming a heparin-PF4 complex that serves as an antigen, triggering the production of immunoglobulin G (IgG) antibodies (HIT antibodies) and eliciting an immune response. These HIT antibodies form immune complexes with heparin-PF4 complexes, and the Fc portion of the HIT antibody bridges with FcγIIa (IgG) on platelets, triggering strong platelet activation and aggregation. In addition, platelet activation leads to the production of microparticles, accelerating aggregation and triggering systemic thrombosis associated with excessive production of thrombin [1]. Consequently, venous thrombosis, arterial thrombosis, and death occur in 17–55%, 3–10%, and approximately 5–10% of patients with HIT, respectively [2]. In patients with acute HIT who require cardiovascular surgery, surgery should be delayed until HIT antibodies disappear. If delaying surgery is not feasible, intraoperative heparin after treatment with preoperative or intraoperative plasma exchange, intraoperative anticoagulation with argatroban, or intraoperative heparin in combination with a potent antiplatelet agent is recommended. In patients with suspected HIT, surgery should be delayed until the results of HIT antibodies come back [5]. However, there is no consensus regarding the protocol for intraoperative anticoagulation in patients undergoing cardiovascular surgery. In this case, the potential cause of the drastic decrease in the platelet counts was IABP. Although heparin solution was administered for patency of the arterial line, it was also initiated to treat deep venous thrombosis as heparin is routinely used as an intraoperative anticoagulant. The results of HIT antibodies came back positive during the surgery, heparin was discontinued, and argatroban was used postoperatively. Aspirin was also initiated on postoperative day 1 as the standard protocol of the iLVAD postoperative course. HIT occurs in approximately 0.3–5% of patients receiving heparin and in a high percentage (8.4 − 20%) of patients with an iLVAD. This high prevalence of HIT in patients with iLVAD is conceivably due to more commonly having prior exposure to heparin than other patients undergoing cardiotomy [3]. In a study by Koster et al., patients diagnosed with HIT after iLVAD implantation demonstrated a markedly higher incidence of severe thromboembolism and tended to have higher mortality than those diagnosed with HIT prior to iLVAD implantation [3]. Patients who develop HIT in the perioperative period of iLVAD implantation, as with our patient, need to be diagnosed earlier and treated carefully. Treatment for HIT requires alternative anticoagulant therapy (often with the antithrombin agent argatroban) followed by conversion to warfarin once the patient’s systemic condition has stabilized. In patients with HIT, initiation of warfarin causes a decrease in protein C earlier than that with prothrombin, which can create a hyperthrombogenic state. Linkins et al. recommend not initiating warfarin until platelet count improves (typically to 150,000/µL), initiating warfarin with a low dose (maximum 5 mg), and discontinuing thrombin inhibitors after a minimum of 5 days of overlap with warfarin [2]. On the other hand, Chen et al. advocated for the safety and efficacy of initiating warfarin after two consecutive increases in platelet counts, even if the platelet counts do not reach 150,000/µL [6]. In the present case, warfarin was initiated in addition to argatroban on postoperative day 1, but the patient’s platelet count at that time was 91,000/µL and continued to decrease. Although we initiated warfarin at a low dose, we likely should have waited until the platelet count had improved. This early initiation of warfarin resulted in a hyperthrombogenic state and led to multiple arterial and venous thromboses on postoperative day 2. Subsequently, we suspended warfarin and administered argatroban for 2 weeks after the procedure. On postoperative day 8, when the patient’s platelet count reached 130,000/µL, the consumption of platelets was considered to be settled, and coagulation cascades had normalized. We transitioned from argatroban alone to warfarin after more than two rising platelet counts instead of waiting for a platelet count of 150,000/µL because the safety and efficacy of argatroban as an anticoagulant for iLVAD were unknown for the prevention of device thrombosis and re-bleeding. Argatroban contributed to inhibiting thrombin production, leading to inhibition of platelet consumption and normalization of platelet counts, all of these could contribute to the lack of subsequent thromboses in the patient, and he was discharged without any sequelae.

## Conclusion

We encountered a case of HIT type II in which multiple thromboses occurred following iLVAD implantation. Administration of warfarin when platelet count was low resulted in a hyperthrombogenic state and multiple thromboses. If HIT antibodies are detected, it is crucial to perform argatroban monotherapy, with oral anticoagulation therapy with warfarin only initiated when the platelet count showed at least two consecutive increases and reached nearly normal range (150,000/μL is commonly considered as a lower limit of the normal range).

## Data Availability

Not applicable.

## Abbreviations

HIT: Heparin-induced thrombocytopenia
iLVAD: Implantable left ventricular assist device
CE-CT: Contrast-enhanced computed tomography
IgG: Immunoglobulin G 202
